# Aspect angiographique des stries angioïdes compliquées de néeovascularisation choroïdienne bilatérale

**DOI:** 10.11604/pamj.2015.21.200.6239

**Published:** 2015-07-15

**Authors:** Alami Fadoua, Berraho Amina

**Affiliations:** 1 Université Mohammed V Souissi, Service d'Ophtalmologie B Hôpital des Spécialités CHU Rabat, Maroc

**Keywords:** Stries angioïdes, néovaisseaux choroïdiens, angiofluographie, angioid streaks, choroidal neovascularization, angio fluorography

## Image en médecine

Il s'agit d'un patient de 67 ans, hypertendu équilibré sous traitement qui consulte pour un syndrome maculaire avec baisse marquée de l'acuité visuelle bilatérale. L'examen ophtalmologique trouve une acuité visuelle corrigé de 4/10 P2 en œil droit, et 1/10 avec scotome centrale œil gauche, un segment antérieur normal en œil droit-gauche, au fond d’œil on note l'aspect de stries partant de la papille en œil droit-gauche compliqué de néovaisseaux choroïdiens juxta-fovéolaires bilatérale. En angiofluorographie, on note une hyperfluorescence inhomogène dès les temps précoces, maximale aux temps intermédiaires avec l'imprégnation des néo-vaisseaux choroïdiens juxta-fovéolaires dès les temps précoces et diffusion tardive en œil droit-gauche (A,B,C). La gravité des stries angioides est la néovascularisation choroïdienne maculaire juxta-fovéolaire et rétro-fovéolaire responsable de malvoyance de manière inéluctable quelque temps après sa survenue d'où l'intérêt de surveiller le patient de façon régulier par une angiographie à la fluorescéine pour un diagnostic précoce et une meilleure prise en charge.

**Figure 1:**
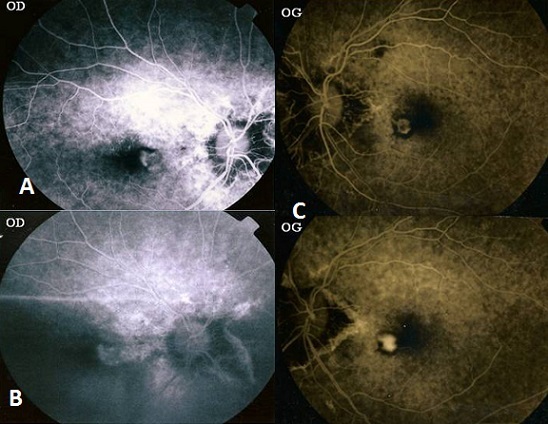
(A): cliché angiographique au temps précoce montre une imprégnation des NVC juxta-fovéolaires au niveau de l’œil droit; (B): NVC juxta-fovéolaires de l’œil droit au temps tardif; (C): cliché angiographique de l’œil gauche montrant une imprégnation des néovaisseaux choroïdiens juxta-fovéolaires au temps précoce (haut) puis au temps tardif (bas)

